# Structural classification by the Lipase Engineering Database: a case study of *Candida antarctica *lipase A

**DOI:** 10.1186/1471-2164-11-123

**Published:** 2010-02-19

**Authors:** Michael Widmann, P Benjamin Juhl, Jürgen Pleiss

**Affiliations:** 1 Institute of Technical Biochemistry, University of Stuttgart, Allmandring 31, 70569 Stuttgart, Germany

## Abstract

**Background:**

The Lipase Engineering Database (LED) integrates information on sequence, structure and function of lipases, esterases and related proteins with the α/β hydrolase fold. A new superfamily for *Candida antarctica *lipase A (CALA) was introduced including the recently published crystal structure of CALA. Since CALA has a highly divergent sequence in comparison to other α/β hydrolases, the Lipase Engineering Database was used to classify CALA in the frame of the already established classification system. This involved the comparison of CALA to similar structures as well as sequence-based comparisons against the content of the LED.

**Results:**

The new release 3.0 (December 2009) of the Lipase Engineering Database contains 24783 sequence entries for 18585 proteins as well as 656 experimentally determined protein structures, including the structure of CALA. In comparison to the previous release [1] with 4322 protein and 167 structure entries this update represents a significant increase in data volume. By comparing CALA to representative structures from all superfamilies, a structure from the deacetylase superfamily was found to be most similar to the structure of CALA. While the α/β hydrolase fold is conserved in both proteins, the major difference is found in the cap region. Sequence alignments between both proteins show a sequence similarity of only 15%. A multisequence alignment of both protein families was used to create hidden Markov models for the cap region of CALA and showed that the cap region of CALA is unique among all other proteins of the α/β hydrolase fold. By specifically comparing the substrate binding pocket of CALA to other binding pockets of α/β hydrolases, the binding pocket of *Candida rugosa *lipase was identified as being highly similar. This similarity also applied to the lid of *Candida rugosa *lipase in comparison to the potential lid of CALA.

**Conclusion:**

The LED serves as a valuable tool for the systematic analysis of single proteins or protein families. The updated release 3.0 was used for the evaluation of α/β hydrolases. The HTML version of the database with new features is available at http://www.led.uni-stuttgart.de and provides sequences, structures and a set of analysis tools including phylogenetic trees and HMM profiles

## Background

Lipases (triacylglycerol hydrolases E.C. 3.1.1.3) are a versatile group of enzymes which catalyze the hydrolysis or synthesis of a broad range of water insoluble esters.

They belong to the class of α/β-hydrolases which also contains esterases, acetylcholinesterases, cutinases, carboxylesterases and epoxide hydrolases. Despite their high diversity in sequence and function, the α/β-hydrolases share a common architecture, the α/β-hydrolase fold [2] and conserved active site signatures, the GxSxG and GxDxG motifs [3,4]. Two conserved features found in all α/β-hydrolases are the active site, consisting of the catalytic triad of S-D(E)-H, and the oxyanion hole. Depending on the amino acids involved in forming the oxyanion hole, the enzymes can be classified into three classes, the GGGX-, GX-, and the Y-class [3]. The Lipase Engineering Database (LED) [5] is a resource of fully and consistently annotated superfamilies and homologous families of α/β hydrolases including multisequence alignments of all families. The curation and annotation process for the LED is supported by DWARF [1], an inhouse data warehouse system for protein families. The LED is accessible by a web interface at http://www.led.uni-stuttgart.de. It can be browsed on the level of families, organisms, or structures, and BLAST searches can be performed against all sequence entries.

Prominent members of the α/β hydrolases are the two lipases from *Candida antarctica*. Lipase B is a versatile and well characterized biocatalyst in many organic syntheses and biotransformations [6-8] and shows a low sequence similarity to other α/β hydrolases. The second lipase from *Candida antarctica*, lipase A (CALA), shows a number of unique biocatalytic properties among hydrolases, e.g. high thermostability and stability at acidic pH ranges and the acceptance of tertiary and sterically hindered alcohols [9]. CALA also has a low sequence similarity to other members of the α/β hydrolase fold including lipase B. Therefore it was not included in previous versions of the LED. Only after its structure was recently determined [10], a detailed analysis of its structure identified CALA unambiguously as a member of the α/β hydrolase family. However, in this structure the active site is not accessible to a substrate, therefore the molecular details of substrate binding or the existence of a possible lid are still elusive.

## Results

### Database content and layout

Release 3.0 of the Lipase Engineering Database (LED) contains 18585 proteins with 24783 sequence and 656 structure entries of which about 14000 protein and 489 structure entries are new. Six new homologous families and one new superfamily (the "Candida antarctica lipase A like" superfamily) have been added to the LED in the update process. Seed sequences for the new "Candida antarctica lipase A like" superfamily (LED identifier: abH38) included the sequence from the resolved crystal structure (gi: 160286179) and three sequences of homologous lipases from other organisms (*Kurtzmanomyces sp. - *gi: 20429169, *Malassezia furfur *- gi: 73765555, *Ustilago maydis *- gi: 71018653) which showed high sequence similarity to *Candida antarctica *lipase A (CALA) [10]. Most of the sequences of the superfamily abH38 have already been assigned to a common protein family in other protein family databases. In the Pfam database [11] they are included in the LIP (PF03583) family, in the InterPro database [12] in the family IPR005152, and in the ESTHER [13] database in the Fungal-Bact_LIP family. Because we included only sequences of high sequence similarity to guarantee a good alignment of all sequences of individual families, especially of active site residues, our family abH38 contains less sequences than the respective protein families of the other databases. The four largest superfamilies in release 3.0 contain 50% of all proteins in the LED: The "Cytosolic Hydrolases" superfamily (LED identifier: abh08) with 3188 proteins, containing epoxide hydrolases and haloalkane dehalogenases, the "Carboxylesterases" superfamily (LED identifier: abh01) with 2998 proteins, containing a wide range of carboxylesterases, such as acetylcholine esterases and bile salt activated lipases, the "Moraxella lipase 2 like" superfamily (LED identifier: abh04) with 1781 proteins containing mainly lipases and carboxylesterases, and the "Microsomal Hydrolases" superfamily (LED identifier: abh09) with 1336 proteins, containing microsomal epoxide hydrolases and peptidases. The "Cytosolic Hydrolases" and "Microsomal Hydrolases" superfamilies (abh08 and abh09) belong to the GX-class of α/β hydrolases, the "Carboxylesterases" and "Moraxella lipase 2 like" superfamilies (abh01 and abh04) belong to the GGGX-class of α/β hydrolases.

### Candida antarctica lipase A protein family

**Figure 1:**
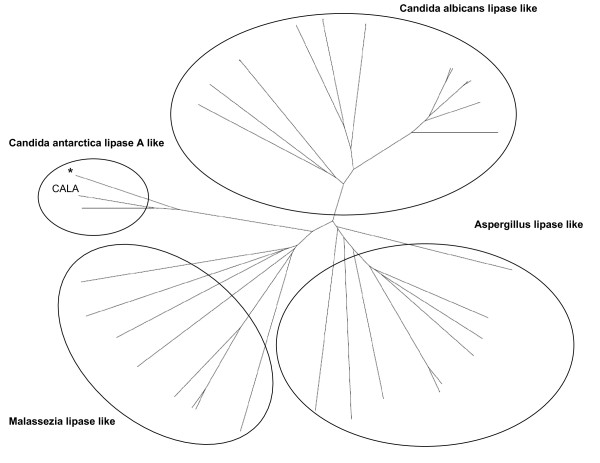
**Phylogenetic tree of the "Candida antarctica lipase A like" superfamily**. The superfamily consists of 4 homologous families based on sequence similarity. The sequence of *C. antarctica *lipase A is indicated.

**Figure 2:**
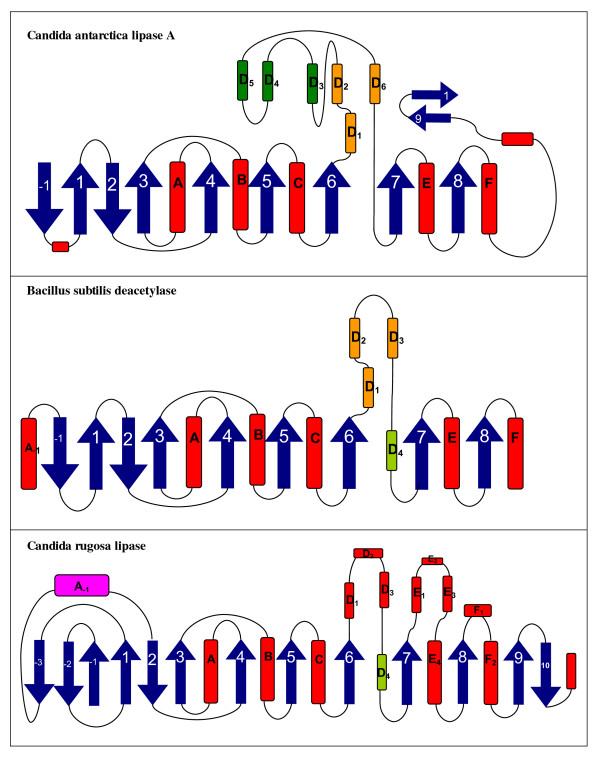
**Topology diagrams of *Candida antarctica *lipase A, *Bacillus subtilis *deacetylase and *Candida rugosa *lipase**. The shared cap region between *C. antarctica *lipase A and *B. subtilis *deacetylase is colored orange. The additional 3 α-helices of the cap region in CALA are labelled D_3_, D_4 _and D_5 _and colored in dark green. The C-terminal, presumably lid forming β-strands of CALA are labelled 9 and 10. The lid forming α-helix of *C. rugosa *lipase is labelled A_-1_. PDB identifiers for the structures are 2VEO (*Candida antarctica *lipase A), 1L7A (*Bacillus subtilis *deacetylase), and 1CRL (*Candida rugosa *lipase).

**Figure 3:**
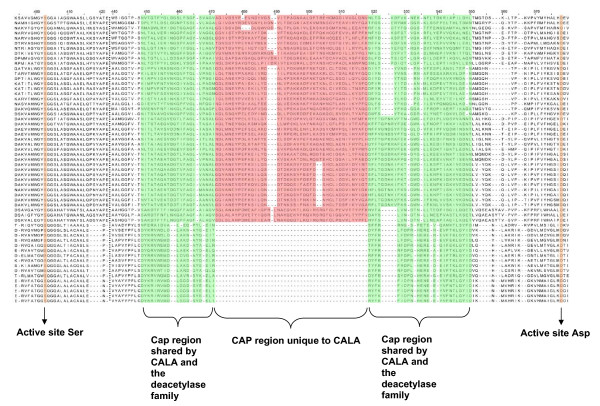
**Multisequence alignment between the "Candida antarctica lipase A ike" and "Deacetylases" superfamilies**. Shown is an excerpt of the alignment containing the active site Ser and Asp and the cap region. The cap region of both superfamilies is colour coded: shared regions green; the additional 3 α-helices of the "Candida antarctica lipase A like" superfamily red. Columns containing active site residues are coded orange.

The "Candida antarctica lipase A like" superfamily contains one crystal structure and 39 sequences, assigned to 32 proteins. They were grouped into four homologous families based on sequence similarity. Homologous families were named by the organism of origin of the dominating proteins in the respective family (Figure 1): The "Candida antarctica lipase A like" homologous family consisting of Lipase A from *C. antarctica*, the "Malassezia lipase like" homologous family consisting entirely of lipases and esterases from *Malassezia globosa *or *Malassezia furfur*, the "Candida albicans lipase like" homologous family consisting of various isoforms of the secretory lipase from *Candida albicans*, and the "Aspergillus lipase like" homologous family consisting mainly of hypothetical or putative lipases, mostly from *Aspergillus*. All 32 proteins are from organisms belonging to the subkingdom Dikarya of the kingdom Fungi. 12 proteins are classified as either lipases or esterases in GenBank [14] while 20 proteins are classified as putative or hypothetical. The only structure entry in this superfamily is from the recently resolved crystal structure of CALA [10]. Based on the structure of the oxyanion hole, CALA can be classified as a Y-class lipase, and Tyr 93 was identified as the oxyanion hole forming amino acid. A structural comparison with other structures from the LED identified a structure from the deacetylase superfamily (LED identifier: abH26) as most closely related. A detailed structural alignment of CALA (PDB: 2VEO) with the structure of the *Bacillus subtilis *deacetylase (PDB: 1L7A) from the deacetylase superfamily showed a superimposition of the common α/β hydrolase fold including the catalytic triad (2VEO: Ser184, Asp334, His366; 1L7A: Ser181, Asp269, His298), despite having a low overall sequence identity of only 15%. Structural differences between the two structures are found in the cap and the C-terminal region. The cap region, located between β-strands 6 and 7, often confers substrate specificity or additional functions to the enzyme [15]. In the case of CALA, the cap region is involved in forming the tunnel like binding site for the acyl moiety [10]. For *B. subtilis *deacetylase, the cap region partially shields the active site from the solvent [16]. The cap region of CALA consists of six α-helices, while the cap region of *B. subtilis *deacetylase consists of only four α-helices (Figure 2). Three α-helices in both proteins are found at identical positions. CALA shows an insert of three additional α-helices after the first two conserved α-helices and is missing the last α-helix of the cap present in *B. subtilis *deacetylase (Figure 2). In order to identify residues in the cap region which are conserved between the "Candida antarctica lipase A like" and "Deacetylases" superfamilies, a multisequence alignment of each family was performed. The two family alignments were aligned using a structural alignment of the two protein structures (Figure 3). The alignment demonstrated that despite the high structural similarity, there are no conserved residues in the cap region of both protein families. Two hidden Markov models of the three inserted α-helices of CALA and the α-helices shared by both proteins were created and used to search against all other protein families of the LED. No sequences with a significant similarity were found in the entire database, demonstrating that the sequence of the cap region of the "Candida antarctica lipase A like" superfamily is unique.

**Figure 4:**
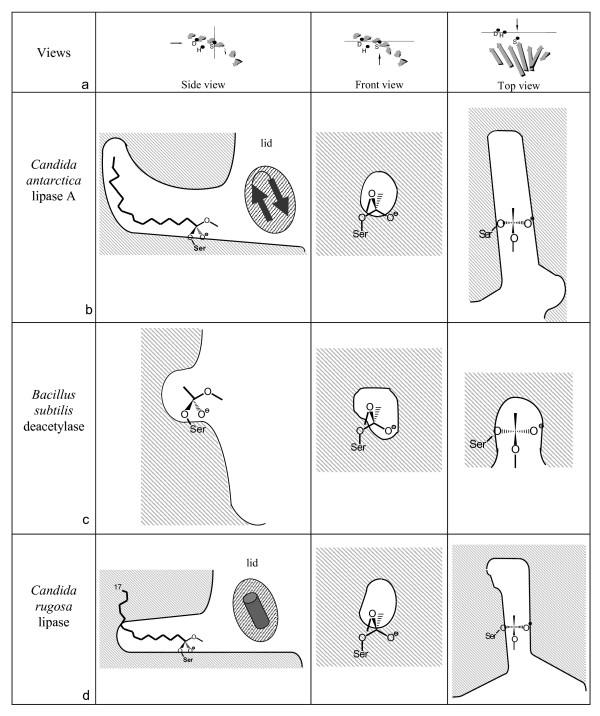
**Shape of the binding site of *Candida antarctica *lipase A, *Bacillus subtilis *deacetylase and *Candida rugosa *lipase**. (a) Orientation of the cross-sections which are planes perpendicular to the paper plane and indicated by a straight line. The direction of the view is indicated by an arrow. Shape of the binding sites is displayed in side, front and top view for (b) *Candida antarctica *lipase A, (c) *Bacillus subtilis *deacetylase, and (d) *Candida rugosa *lipase. A model of the acyl moiety of the substrate is displayed, the alcohol moiety is not shown for clarity. The position of the lid in a closed state for *Candida antarctica *lipase A and *Candida rugosa *lipase is indicated.

In comparison to the *B. subtilis *deacetylase, CALA has two additional β-strands (9 and 10) in the C-terminal region (Figure 2). They are positioned directly above the active site and prevent a direct access of the substrate to the active site. We assume that the β-strands 9 and 10 perform a lid like function for CALA since movement of the two β-strands would allow substrate access to the active site of CALA from a similar direction as for the *B. subtilis *deacetylases [16]. A comparison of the substrate binding sites of both proteins showed that the alcohol binding site is similar in both proteins and provides ample space for alcohol moieties of substrates (Figure 4). Therefore, both proteins are expected to accept a variety of bulky alcohols. The binding sites for the acyl moieties are highly different. CALA has a long, tunnel like binding site, while the *B. subtilis *deacetylase has a small cavity which is part of a cleft on the protein surface. Therefore, the acyl moieties of the substrates are expected to differ significantly between both enzymes. CALA is expected to accept mediumt to long chain fatty acids, while the *B. subtilis *deacetylase is limited to short-chain acyl moieties. Thus, despite the overall similarity between CALA and the *B. subtilis *deacetylase, the acyl binding site is fundamentally different.

However, the binding site of CALA shows surprising similarity to another lipase, *Candida rugosa *lipase (CRL). For CRL, two different structural confirmations have been resolved, an open conformation (1CRL) [17], and a closed conformation (1TRH) [18] where the lid of CRL is blocking the substrate access to the active site. CRL has a cap region between β-strands 6 and 7, consisting of four α-helices (Figure 2). The substrate binding site of CRL consists of a long tunnel for the acyl moiety of the substrate and provides ample space for the alcohol moiety of the substrate (Figure 4). Despite having a lower overall structure similarity to CALA than the *B. subtilis *deacetylase, the binding sites of CALA and CRL are highly similar (Figure 4). Both provide space for large, bulky alcohol moieties of the substrate and have a tunnel like binding site for the acyl moiety. Both proteins posses a lid which covers the active site and prevents direct access to the substrate binding site in its closed state. The lid of CRL lipase is formed by a α-helix between β-strands 1 and 2 and is located in the N-terminal region while the putative lid in CALA is formed by the two C-terminal β-strands 9 and 10 (Figure 2).

## Discussion

The LED contains annotated and systematically classified protein families of α/β hydrolases. It has been shown to be a useful tool for the systematic analysis of protein families. Previous work employed the LED and BLAST in order to identify novel enzymes belonging to the α/β hydrolase fold [19,20]. A model for the prediction of protein solubility was developed and refined by performing a comprehensive analysis of the protein families of the LED [21]. A further study involved the systematic analysis of protein families of the LED in regard to the distribution and conservation of functionally relevant rare codons [22].

Since the first release of the LED [3], more than 14000 new α/β hydrolases became available and were integrated in the release 3.0. As a case study for the utility of the highly enriched and annotated database, the newly introduced superfamily of CALA was analysed and compared to other protein structures in the LED. The goal was to characterise the sequence and structure of CALA in comparison to other α/β hydrolases despite its low sequence similarity and to understand the molecular basis of substrate recognition.

While CALA shows structural similarity to the deacetylase family, the substrate specificity of both enzymes differs, which is consistent with the differences observed in the substrate binding sites of both proteins. In contrast, the lipase from *C. rugosa *(CRL), which shows a lower overall structural similarity to CALA, is remarkably similar in regard to the substrate binding site. The structural similarities and differences are in accordance with experimentally observed substrate specificities of the three enzymes. All three proteins have a spacious alcohol binding site. The *B. subtilis *deacetylase accepts a wide variety of bulky substrates like cephalosporin C and xylose [16]. CALA and CRL also accept bulky substrates, ranging from primary alcohols to sterically hindered secondary alcohols and even tertiary alcohols [23,24].

The tunnel like binding site of CALA allows the enzyme to accept esters of long chain fatty acids [23,25]. The similar tunnel like acyl binding site of CRL also accepts fatty acids up to a chain length of 18 [25]. In contrast, the small acyl binding site of the *B. subtilis *deacetylase is unable to accept large acyl groups and is restricted towards acetyl moieties [16]. Experimentally, CALA and CRL have been shown to display interfacial activation [26,27]. While a lid in CRL has been localized and the open and closed form of CRL has been crystallized ([17,18]), the lid function of the β-strands 9-10 in CALA remains to be experimentally verified. However, the similarities to CRL suggest a substrate access involving the movement of β-strands 9-10.

## Conclusions

The analysis of the newly introduced protein family of *Candida antarctica *lipase A demonstrates the strength of our database approach by providing a large set of protein families which share a common protein fold despite an overall low sequence similarity. By combining both, structural and sequential information of a large number of proteins a thorough analysis and classification of proteins of interest is made possible. The Lipase Engineering Database (LED) is online accessible at http://www.led.uni-stuttgart.de. All information on families of sequence and structure data, as well as alignments, phylogenetic trees, and family-specific profiles can be accessed by manual download.

## Methods

### Structural comparisons and alignments

Comparison of structures were carried out using DALI [28]. The structure of CALA was compared against 28 representative structures from all superfamilies. To identify the most closely related superfamilies, only structures which could be aligned to more than 50% of the residues of CALA were considered. Structural alignments of proteins were performed by STAMP [29].

For superfamilies which share a close structural relationship but a low overall sequence identity, a two step strategy was used in order to obtain a more significant multisequence alignment. First, a multisequence alignment for each of the two superfamilies was carried out separately. Then a structural alignment, between reference structures from each protein family was performed using STAMP [29]. The multisequence alignments where then aligned against the structure from their respective protein family.

### Sequence analysis

Multisequence alignments for all protein families were generated using ClustalW [30] with a gap opening and extension penalties of 10 and 0.2, respectively. Hidden Markov models were created using HMMER [31].

### Database model

The implemented data model is based on Firebird [32] and is based on the previously published [1] data model (Figure S1, Additional file 1). Protein families are organised on the level of homologous families and superfamilies based on their sequence similarity. The database is updated by an automated Perl [33] script. It performs a BLAST [34] search against the current version of the non-redundant sequence database an NCBI [14] for each sequence entry with an E-value cut-off of 10^-50^. Crystal structure information referring to new sequence entries is updated as well. New sequence and structure entries are assigned to homologous families and superfamilies based on sequence similarity. New families which consisted of only one putative protein entry where not included. Annotation information of residues is either taken directly from the according GenBank entry or is transferred to new sequences using the DWARF graphical user interface. Annotation information is then transferred to the newly integrated sequences.

## Authors' contributions

MW performed all analyses regarding CALA and drafted the manuscript. MW and BJ performed the update of the database and the manual curation process. JP supervised the project and finalized the manuscript. All authors read and approved the final manuscript.

## Supplementary Material

Additional file 1 **Microsoft Word 2003**. Conceptual data scheme for the LED using Logical Data Structure (LDS) notation. Click here for file
