# Supplementary material for: Structural classification by the Lipase Engineering Database: a case study of Candida antarctica lipase A
Source: BMC Genomics. 2010 Feb 19;11:123. doi: 10.1186/1471-2164-11-123 (PMC2841678; doi:10.1186/1471-2164-11-123)
Supplement: Additional file 1 — Microsoft Word 2003. Conceptual data scheme for the LED using Logical Data Structure (LDS) notation. [file 1471-2164-11-123-S1.DOC]

**Structural classification by the Lipase Engineering Database: a case study of *Candida antarctica* lipase A**

**Michael Widmann, P. Benjamin Juhl, and Jürgen Pleiss**

**Institute of Technical Biochemistry, University of Stuttgart,**

**Allmandring 31, 70569 Stuttgart, Germany**

**Additional file 1**


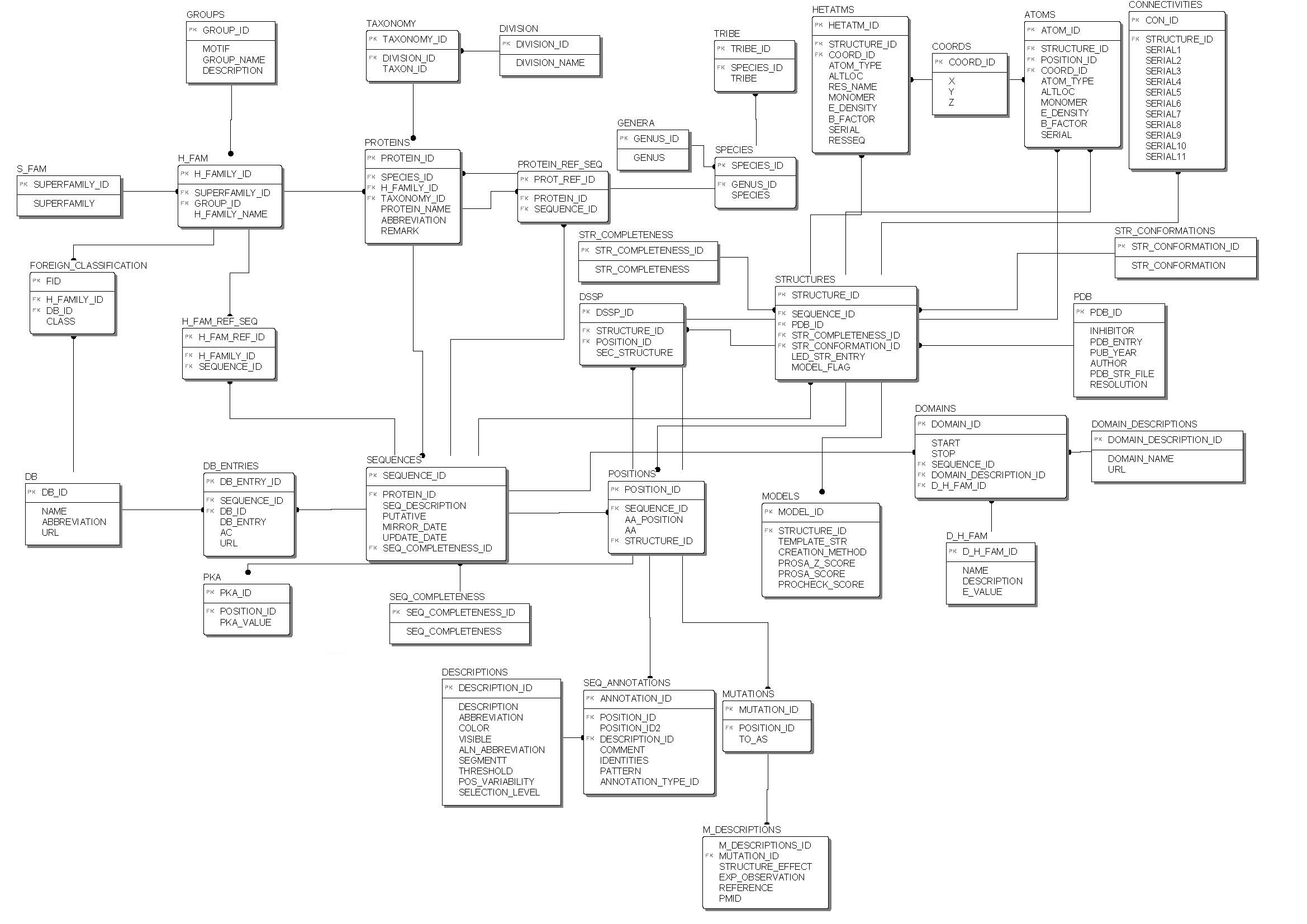


**Figure S1 - Conceptual data scheme for the LED using Logical Data Structure (LDS) notation**

Each database table is represented by a separate table. Primary key attributes are displayed in the header of the respective table.
